# The first *Cyclospora cayetanensis* lineage A genome from an isolate from Mexico

**DOI:** 10.1186/s12864-024-10163-y

**Published:** 2024-03-05

**Authors:** Monica Santin, Aleksey Molokin, Guadalupe E. Orozco-Mosqueda, Sonia Almeria, Jenny Maloney

**Affiliations:** 1 Environmental Microbial and Food Safety Laboratory, Agricultural Research Service, Department of Agriculture, 20705 Beltsville, MD USA; 2 Hospital Infantil de Morelia Eva Sámano de López Mateos, Servicio de Salud de Michoacán, 58020 Morelia, Michoacán México; 3 U.S. Food and Drug Administration, Center for Food Safety and Applied Nutrition, Office of Applied Research and Safety Assessment, Division of Virulence Assessment, 20708 Laurel, MD USA

**Keywords:** *Cyclospora cayetanensis*, Genome, Mexico, Lineage A

## Abstract

**Background:**

*Cyclospora cayetanensis* is a protozoan parasite that causes intestinal illness in humans worldwide. Despite its global distribution, most genomic data for *C. cayetanensis* has been obtained from isolates collected in the United States, leaving genetic variability among globally distributed isolates underexplored.

**Results:**

In the present study, the genome of an isolate of *C. cayetanensis* obtained from a child with diarrhea living in Mexico was sequenced and assembled. Evaluation of the assembly using a lineage typing system recently developed by the Centers for Disease Control and Prevention revealed that this isolate is lineage A.

**Conclusions:**

Given that the only other whole genome assembly available from Mexico was classified as lineage B, the data presented here represent an important step in expanding our knowledge of the diversity of *C. cayetanensis* isolates from Mexico at the genomic level.

**Supplementary Information:**

The online version contains supplementary material available at 10.1186/s12864-024-10163-y.

## Background

*Cyclospora cayetanensis* is an obligate intracellular protozoan parasite of the phylum Apicomplexa that can cause severe intestinal illness in humans. Infections with this parasite have become a major public health and food safety concern. Cyclosporiasis is an anthroponotic disease meaning that source of infection for human beings is another human with no known intermediate or reservoir hosts. People acquire cyclosporiasis via the fecal-oral route by consuming food or water contaminated with sporulated oocysts [1]. Fresh produce that is consumed raw such as berries, herbs, or leafy greens have all been linked to outbreaks of *C. cayetanensis*, and the parasite has been detected in fresh produce worldwide [1, 2]. The parasite has also been frequently detected in water, which is considered a vehicle of transmission [1–3]. Likewise *Cyclospora* infection has been reported in humans worldwide with *C. cayetanensis* being considered endemic in most tropical and subtropical countries [2]. In the United States (U.S.), recurring outbreaks of cyclosporiasis linked to both domestic and imported produce have made *C. cayetanensis* an emerging public health concern, and when imported produce is implicated in infection, the country of origin of the isolate can be difficult to conclusively demonstrate [1, 2].

*Cyclospora cayetanensis* has been recognized as endemic in Mexico and countries of Central and South America. Yet molecular epidemiology data is sparse from these regions. This is especially true for whole genome sequence data with only five whole genome assemblies available from isolates from these regions including a single isolate from Mexico and four from Guatemala (Additional file 1). Currently, most whole genome sequence data for *C. cayetanensis* come from isolates collected in the U.S. However, data from isolates collected in other regions, especially those regions where cyclosporiasis is endemic, are needed to understand the epidemiology and improve molecular tracking tools of this important human pathogen.

In the present study, the genome of a *C. cayetanensis* isolate, obtained from a child with diarrhea living in Mexico, was sequenced using Illumina MiSeq. The assembly from this isolate was assessed for lineage markers as recently described by the Centers for Disease Control and Prevention (CDC) to determine its lineage with a method that uses similarity scores between representative loci for lineage assignments [4]. Comparisons between this isolate and other available *C. cayetanensis* whole genome assemblies from National Center for Biotechnology Information (NCBI) were also made.

## Methods

### Source of isolate

The isolate of *C. cayetanensis* was obtained from a 10-year-old male patient experiencing diarrhea seeking medical attention at the outpatient clinic of the Pediatric Hospital in Morelia (Mexico) in October 2016. Stool specimens were submitted for standard ova and parasite examination that included direct smear and concentration using Sheather and Ritchie standard methods. Oocysts of *C. cayetanensis* were identified and the stool sample was stored in 2.5% (w/v) aqueous potassium dichromate solution at room temperature. The de-identified sample was sent to the Environmental Microbial and Food Safety Laboratory (ARS-USDA) in Beltsville, MD for sequencing. Ethics approval was obtained from the Institutional Review Board at the Hospital Infantil de Morelia Eva Sámano de López Mateos with register number HIM/LMP/15/2020.

### Oocyst purification and DNA extraction

The preservative, potassium dichromate, K_2_Cr_2_O_7_, was removed by centrifugation (1300xG) at 4 °C for 10 min and decanting of the supernatant. The resulting approximately 5 ml pellet was resuspended in distilled water, centrifuged (1300xG) at 4 °C for 10 min, and the supernatant was decanted. The pellet was then resuspended and the oocysts were cleaned and concentrated using cesium chloride density gradient centrifugation as described [5]. Oocysts collected from the gradient were treated with 10 µl of a 1× of antibiotic–antimycotic solution (Invitrogen, Waltham, MA) and incubated overnight at 4 °C. The supernatant was aspirated, pellet resuspended in distilled water, washed by centrifugation (1300xG) at 4 °C for 10 min, and supernatant decanted. Then, oocysts were treated with 1 mL of 3% sodium hypochlorite solution for 10 min at 4 °C. Bleach was removed by centrifugation (1300xG) at 4 °C for 10 min and decanting of the supernatant. The resulting pellet was resuspended in distilled water, centrifuged (1300xG) at 4 °C for 10 min, and supernatant decanted.

After cleaning, oocysts were quantified using a Zeiss Axioskop microscope equipped with epifluorescence and an FITC- Texas Red™ dual wavelength filter that aided in visualization of oocysts by autofluorescence. Counting was performed with a hemocytometer in triplicate. The estimated total number of oocysts of *C. cayetanensis* was 1 × 10^6^. Total genomic DNA was extracted from the 1 × 10^6^ oocysts using the DNeasy Tissue Kit (Qiagen, Valencia, CA) following the manufacturer’s instructions with minor modifications. Modifications included an overnight incubation with proteinase K and a final elution with 100 µl of AE buffer. DNA concentration was determined by Qubit (Invitrogen, Waltham, MA) with a total yield of 14.04 ng.

### Illumina library preparation and sequencing

One nanogram of genomic DNA was used for whole genome sequencing using the Nextera XT DNA Prep kit (Illumina, San Diego, CA). Library quantification was performed via Qubit (Invitrogen, Waltham, MA), and fragment size was estimated using a 4200 TapeStation System (Agilent, Santa Clara, CA). The final library was sequenced using an Illumina MiSeq (Illumina, San Diego, CA) with v3 600 cycle sequencing kit (2 × 300 bp) following the manufacturer’s instructions.

### Genome assembly and analysis

FASTQ read pairs were adapter trimmed, length filtered (minlength = 75), and merged using bbduk and bbmerge from the bbtools software package v38.79 (options: rem, k = 62, extend2 = 50, ecct, vstrict, mininsert = 75) [6]. Reads were mapped to reference genomes using minimap2 v2.24 [7]. *De novo* assembly was performed using SPAdes v3.15.5 (options: --careful, --cov-cutoff 5) [8]. Assembly re-scaffolding relied on the chromosome_scaffolder.sh script that is bundled with the MaSuRCA assembler v4.6.1 (option --nb enabled) [9]. *De novo* gene prediction was performed using Genemark_ES v4.71, and reference genes were aligned to the *de novo* assembly using the annotation transfer tool, Liftoff v1.6.3 (options --polish and --copies) [10, 11]. GTF files containing predictions or annotations were parsed using AGAT v1.2.0 (agat_sp_filter_incomplete_gene_coding_models.pl) to summarize the number of complete and incomplete protein coding genes [12]. A BUSCO analysis was performed to estimate genome completeness of draft assemblies using BUSCO v5.4.7 and the OrthoDB v10/Coccidia dataset [13]. Synteny between assemblies was assessed using Mauve [14]. Whole genome phylogenetic distances were calculated using phylonium v1.7 (options --2pass --complete-deletion) [15]. Neighbor-joining phylogeny was built using the mattools nj command and tree formatting and plotting was performed using the R packages phangorn v2.11.1, ggtree v3.8.2, and ggplot2 v3.4.3 [16–18]. Gene marker phylogenetic analyses were performed by extracting and concatenating three loci from all available whole genome *C. cayetanensis* assemblies on NCBI in the following order: partial apicoplast genome (∼ 18 kb), putative cysteine proteinase (∼ 1.5 kb), partial polyamine-modulated factor 1-binding protein 1 (∼ 2.5 kb). These loci were selected because they have been previously shown to support the recently described lineage designations within *C. cayetanensis* [4]. The total concatenated length was ∼ 22 kb. Assemblies that did not contain all three loci were excluded. Concatenated sequences were aligned using Clustal Omega v1.2.4 with default parameters, and the alignment was then imported into MEGA v11.0.11 to generate Maximum Likelihood (ML), neighbor-joining (NJ), and UPGMA trees using 1000 bootstrap replicates [19]. Genome assemblies and raw sequences are available at NCBI under the BioProject PRJNA1045665.

## Results

### Assembly metrics and gene predictions

In this study, the genome of an isolate of *C. cayetanensis* obtained from a human patient in Mexico was sequenced via Illumina MiSeq and given the isolate name USDA_Mex32. To determine if the isolate USDA_Mex32 represents a clonal population, the methods proposed by Barratt et al., (2023) to assess the purity of a strain based on several genotyping loci was used [4]. Based on this analysis, if two or less haplotypes are observed for nuclear loci and only one haplotype is observed for mitochondrial loci, the assumption of a strain pure isolate can be made [4]. Using these criteria, USDA_Mex32 represents a strain pure isolate with the patient sample likely representing a clonal population.

To assess coverage of the *C. cayetanensis* genome using the USDA_Mex32 reads, Illumina reads were mapped to two reference genomes (Table 1). The two *C. cayetanensis* reference genomes used for comparison, Can-NML:CYC2020-001 (GCA_020976615.1) from Canada and NF1_C8 (GCA_002999335.1) from Nepal, were selected based on their status as most contiguous and most annotated references available at the time of analysis, respectively [20, 21]. Mapping USDA_Mex32 reads to these reference genomes demonstrated a high breadth of coverage, that nearly all reads mapped to reference, and an average read depth of 115.84x and 141.96x (Table 1).

**Table 1:** Mapping Illumina reads of *C. cayetanensis* isolate USDA_Mex32 to *C. cayetanensis* reference genomes

	Isolate ID (GenBank Accession #)	
	Can-NML:CYC2020-001 (GCA_020976615.1)	NF1_C8 (GCA_002999335.1)
Reads mapped (%)	99.3	98.9
Breadth of coverage (%)	99.9	99.6
Average depth (x)	141.96	115.84

Both merged read pairs and unmerged reads of USDA_Mex32 were used to produce a *de novo* assembly with SPAdes. Additionally, the *de novo* assembly was then ordered, oriented, and re-scaffolded with chromosome_scaffolder.sh using the GCA_020976615.1 reference assembly. While the original *de novo* assembly was highly fragmented at over 1,600 scaffolds, that number is reduced to just 277 after scaffolding with the more contiguous reference assembly (Table 2). Re-scaffolding the USDA_Mex32 assembly also improved *de novo* gene prediction. The percent of non-fragmented genes predicted among the assemblies was 75.0% in the *de novo* assembly compared to 96.1% after re-scaffolding (Table 3). Comparing gene content of the assemblies to annotations available from the GCA_002999335.1 assembly showed that of the 5,793 protein-coding genes in the reference genome, 87.3% were found as complete genes in the *de novo* assembly compared to 94.4% in the re-scaffolded assembly. Although more complete protein-coding genes are reported in the re-scaffolded assembly compared to the GCA_002999335.1 assembly, it is important note that gene predictions for USDA_Mex32 may include pseudogenes that could not be identified in the absence of transcriptomic data.

**Table 2:** Comparison of assembly metrics among *C. cayetanensis* isolate USDA_Mex32 *de novo* assembly, USDA_Mex32 re-scaffolded assembly, and reference assembly GCA_020976615.1

	USDA_Mex32 de novo assembly	USDA_Mex32 re-scaffolded assembly	GCA_020976615.1 assembly
No. of scaffolds	1,644	277	313
Total length	44,485,211	44,747,036	44,586,677
Gaps %	0.02	0.70	0.0
N50^*^	176	24	24
L50^†^	75,213	524,769	523,712
Maximum scaffold length	371,830	1,976,689	1,973,156
No. of scaffolds > 50 Kb	282	123	120
Percent of genome in scaffolds > 50 Kb	64.9	97.9	97

^†^ L50 = The length at which scaffolds of equal or greater length comprise 50% of the assembly

^*^N50 = The smallest number of scaffolds whose length sum is equal to 50% of the assembly size

**Table 3:** Comparison of complete and incomplete protein-coding genes among gene predictions in *C. cayetanensis* isolate USDA_Mex32 *de novo* assembly, USDA_Mex32 re-scaffolded assembly, and the annotated assembly GCA_002999335.1

	USDA_Mex32 de novo assembly	USDA_Mex32 re-scaffolded assembly	GCA_002999335.1 assembly*
Incomplete protein-coding genes	1,853	257	385
Complete protein-coding genes	5,551	6,367	5,408
% complete	75.0	96.1	93.4

* Gene predictions were not performed on the reference assembly in this study. These counts were extracted from the official annotation release GFF file of the GCA_002999335.1 assembly where annotations were generated by the NCBI Eukaryotic Genome Annotation Pipeline [20]

### BUSCO analysis

Completeness of the *de novo* and re-scaffolded assemblies was assessed using BUSCO scores from Coccidia lineage BUSCOs. The *de novo* USDA_Mex32 assembly had three times as many fragmented and missing BUSCOs as the re-scaffolded assembly. Additionally, the re-scaffolded assembly was more complete than either the GCA_020976615.1 or GCA_002999335.1 assemblies or the only other genome available from a Mexican isolate, CDC_HCMX (assembly GCA_003945065.1), as assessed via BUSCO scores (Table 4).

**Table 4:** Percent of BUSCOs from OrthoDB Coccidia dataset present in *C. cayetanensis* isolate USDA_Mex32 assemblies generated in this study and three reference assemblies

			Isolate ID (GenBank Accession #)		
	USDA_Mex32 *de novo* assembly	USDA_Mex32 re-scaffolded assembly	Can-NML:CYC2020-001 (GCA_020976615.1)	NF1_C8 (GCA_002999335.1)	CDC_HCMX010_16 (GCA_003945065.1)
Complete (%)	95.6	98.6	98.0	98.2	98.2
Fragmented (%)	2.6	0.6	1.0	1.0	1.2
Missing (%)	1.8	0.8	1.0	0.8	0.6

### Assessment of synteny between assemblies of ***C. cayetanensis***

Syntenty between USDA_Mex32 and GCA_020976615.1 was assessed using Mauve, which identifies conserved segments between genomes free from rearrangements referred to as Locally Collinear Blocks (LCBs) [14]. Several sizable blocks of synteny were observed between the two assemblies with the largest block being comprised of ∼ 2 Mb and 158 LCBs present (Additional File2). Synteny between USDA_Mex32 and GCA_003945065.1, which represents the only other genome assembly from an isolate from Mexico, was also assessed. Much less collinearity between these two genomes was observed with 333 LCBs present (Additional File3).

### Phylogenetic analyses

**Fig. 1:**
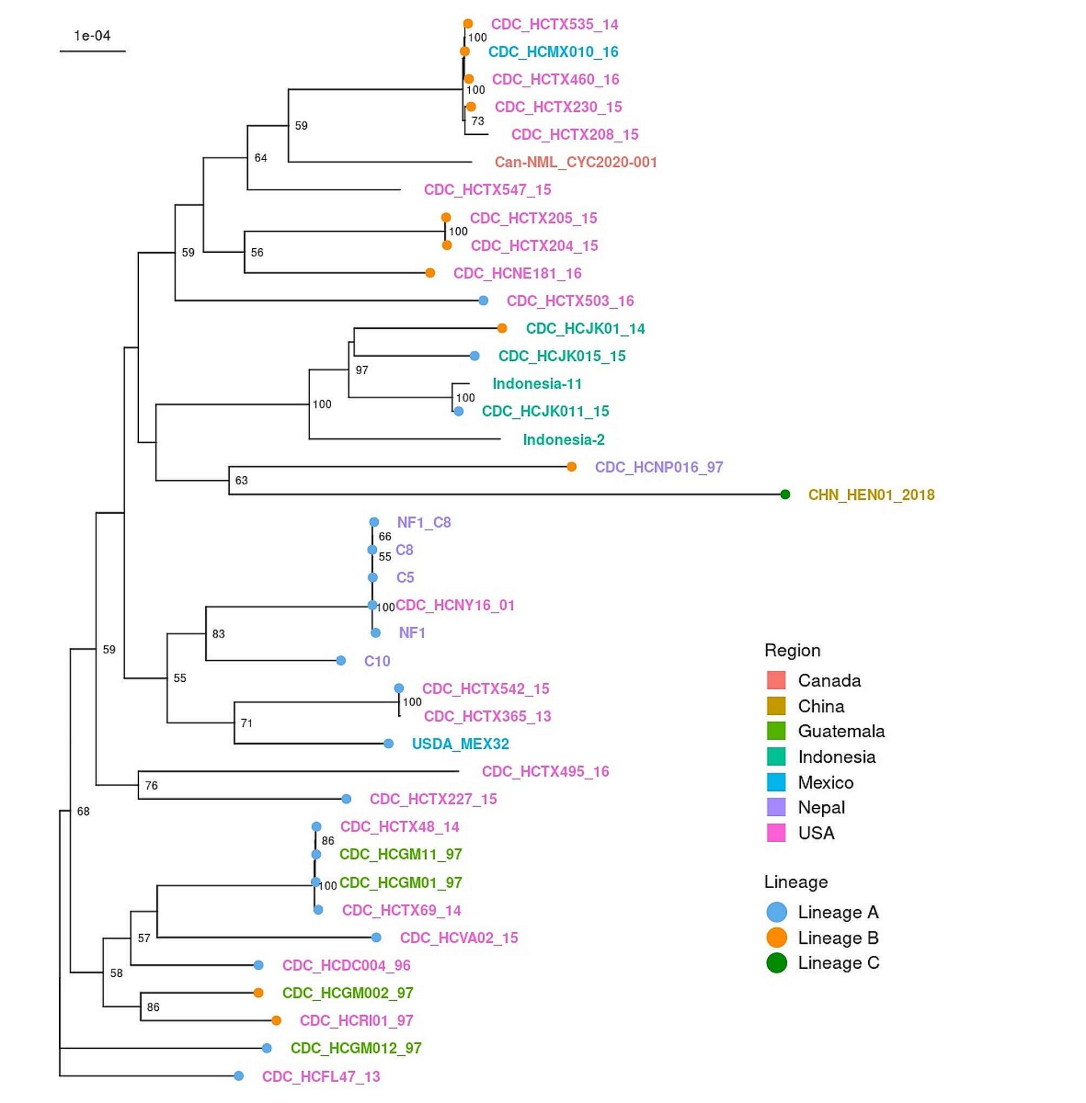
Whole genome phylogeny of USDA_Mex32 isolate and 38 publicly available *C. cayetanensis* assemblies. Color of isolate name indicates country of origin, and, if present, color of branch tip indicates lineage assignments reported by Barratt et al. (2023). Phylogenetic distances were calculated using phylonium, and neighbor-joining tree construction was performed in R. Support values were computed by quartet analysis, and values of > 50 are shown

Recently, a division of *C. cayetanensis* into three genetic lineages, termed A, B, and C, was proposed [4]. To determine the lineage of the USDA_Mex32 isolate, loci used to distinguish lineages were queried in the assembly of USDA_Mex32. According to the lineage classification system, the USDA_Mex32 isolate is lineage A. Additionally, a comparison among all *C. cayetanensis* assemblies available in Genbank and the USDA_Mex32 assembly was performed via construction of a whole genome phylogeny (Fig. 1). In the resulting tree, USDA_Mex32 branched with two isolates from Texas, USA (CDC_HCTX542_15 and CDC_HCTX365_13). Of these two isolates, CDC_HCTX542_15 is listed as lineage A in the CDC typing system, while CDC_HCTX365_13 was not included in the lineage analysis [4]. USDA_Mex32 and these two Texas isolates shared a branch with five isolates from Nepal (NF1, C8, NF1_C8, C5, and C10) and one isolate from New York, USA (CDC_HCNY16_01), which have also been described as lineage (A) However, USDA_Mex32 is part of a larger clade that includes all but two of the previously described lineage B isolates and the only isolate from China which has been described as lineage C. Only one other whole genome assembly for an isolate from Mexico was available in GenBank at the time of this analysis. This isolate, CDC_HCMX010_16, was described as lineage B according to the CDC typing system and formed a separate clade in our tree that included several other lineage B isolates, but also contained a single lineage A isolate, CDC_HCTX503_16. Notably, all isolates from Indonesia formed a separate clade regardless of their classification as lineage A or (B). While the single Nepalese isolate previously classified as lineage B branched with the only lineage C isolate in the analysis. Additionally, all isolates from Asia are interspersed within the same large clade.

**Fig. 2:**
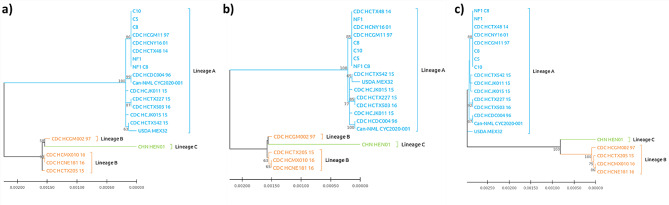
Phylogenetic relationships among *C. cayetanensis* isolates based on concatenated sequences of three lineage typing loci. Analyses were performed using (a) Maximum Likelihood, (b) neighbor-joining, and (c) UPGMA methods with 22,139 bp positions in the final dataset and bootstrapping with 1000 replicates. Only support values of > 50 are shown

To make further comparisons between USDA_Mex32 and other lineage A isolates as well as with isolates of lineages B and C, three loci used for lineage assignment were obtained from genomes in which these loci were present. Twenty genomes comprised the final data set representing isolates from lineages A, B, and C. The remaining available genomes did not contain all loci and were not included in the analysis. Sequences of these loci were concatenated and compared alongside USDA_Mex32 via ML, NJ, and UPGMA analyses. Interestingly, even though all loci included in the analysis have been described as supporting the lineage divisions of A, B, and C, in the present analyses only lineage A isolates consistently cluster together (Fig. 2). Isolates representing lineages B and C do not form distinct clades in the ML and NJ trees and only branch separately in the UPGMA analysis.

## Discussion

Some of the foodborne outbreaks of *C. cayetanensis* in the U.S. and Canada have been linked to fresh produce imported from Mexico [1]. Yet genomic data from Mexico are limited to a single isolate. Given the potential for genomic data to expand our understanding of the epidemiology and biology of *C. cayetanensis*, more data from more isolates from Mexico are needed. In the present study, whole genome sequencing of an isolate from Mexico was performed. The resulting data were used to assess its lineage and compare it to existing whole genome data from 38 isolates from the U.S., Mexico, Guatemala, Nepal, Indonesia, Canada, and China.

To prepare oocysts for sequencing, cleaning and concentration steps including cesium chloride density gradient centrifugation, antibiotic/antimycotic treatment, and washing with bleach were performed. The high coverage of reference genomes and percent of reads mapped to references indicate that the oocyst suspension used in this study was free from contaminants with > 99% of the reads being *Cyclospora*, and the genome of isolate USDA_Mex32 was robustly sequenced (Table 1). Scaffolding the USDA_Mex32 assembly with the most contiguous assembly available for *C. cayetanensis* [21] improved genome completeness in terms of contig order and maximized the number of complete genes (Tables 3 and 4). Assessment of the final USDA_Mex32 assembly metrics demonstrates that these methods can produce an assembly of similar completeness to other available references (Tables 3 and 4). These findings support the use of the isolate preparation, sequencing, and assembly strategy employed here for generation of new *C. cayetanensis* genomes. Additionally, these findings indicate that better reference genomes are needed to assist with reference guided assembly and comparative studies of isolates that have limited genetic material available for sequence generation. It is estimated that the average fecal sample from an infected human would only contain picograms of parasite DNA [22]. Improving our ability to sequence and assemble genomes from isolates with limited quantities of oocysts will be essential in generating the numbers of genomes needed for more robust comparative genomic studies in the future.

Synteny comparisons can provide an overview of the degree of conserved order between genomes. However, little data on the degree of synteny between genomes of *C. cayetanensis* exists. In the present study, comparisons were made between USDA_Mex32 and GCA_020976615.1 and USDA_Mex32 and GCA_003945065.1, and a greater degree of contiguous collinearity was observed between USDA_ Mex32 and GCA_020976615.1 (158 LCBs) than between USDA_Mex32 and GCA_003945065.1 (333LCBs) (Additional Files 2 and 3). These observations are perhaps not surprising as GCA_020976615.1 is both the most contiguous assembly available and was used to re-scaffold the USDA_Mex32 assembly. Additionally, observations of rearrangements or other structural differences between genomes should be interpreted with caution given that all the genomes included in these analyses are highly fragmented. Nevertheless, it is interesting to observe that differences in gene order do exist between genomes of *C. cayetanensis*, and the importance of such differences may become clearer as more contiguous genomes become available for comparison studies.

Strain CDC_HMX010_16 represents the only other whole genome assembly from an isolate from Mexico. CDC_HMX010_16 has been previously classified as lineage B according to the recently developed CDC lineage typing system [4]. This system proposes three genetic lineages named A, B, and C that may represent the species *C. cayetanensis*, *C. ashfordi*, and *C. henanensis*, respectively [4]. The proposal of these lineages as three different species of *Cyclospora* is based on an analysis of thousands of isolates from the U.S. and one isolate from China that found evidence for a lack of gene flow between the proposed lineages indicating the nascent stages of speciation had occurred [4]. Although geographical and temporal associations were noted for lineages A and B in the U.S., no clinical characteristics and limited morphological measurements of only unsporulated oocysts have been described for any of the three lineages [4]. Thus, identification of these three lineages as separate species should perhaps be considered preliminary at this time. Our analyses indicates that USDA_Mex32 is lineage A, making it the only lineage A assembly from Mexico. USDA_Mex32 also branches separately from CDC_HMX010_16 in the whole genome phylogeny produced in this study indicating that meaningful genetic differences may exist among *C. cayetanensis* isolates from Mexico (Fig. 1). Unlike isolates from Indonesia and Nepal which generally branched together by country of origin, isolates from Mexico have a phylogenetic topology that is similar to isolates from the U.S. which also form separate clades. These results support the need for more and better genome level sequence data not only from Mexico but also from other endemic regions to enhance our understanding of the epidemiology of *C. cayetanensis*. Such data will likely have important implications for our ability to improve how outbreak investigations are conducted and may also improve our ability to conduct source tracking and detect *C. cayetanensis*. Additional genomic data can also be used to inform the selection of markers that may be better suited for the development of diagnostic assays targeting identification of *C. cayetanensis* in contamination of fresh produce and water in addition to clinical specimens.

In the present study, isolates previously described as lineages A and B by Barratt et al., 2023 did not strictly segregate into separate clades in the whole genome phylogeny (Fig. 1) [4]. However, the topology observed in the present analysis was similar to what has been observed in other recent analyses employing phylogenies based on concatenated marker genes from multiple loci for genotyping *C. cayetanensis* [23, 24]. In a study designed to develop markers for genotyping *C. cayetanesis* in produce samples, a panel of 52 loci was selected based, in part, on the presence of the loci in the majority of genome sequences [24]. Phylogenetic analyses of these markers and other core chromosomal genes present in available genome sequences did not segregate isolates based on their lineage assignment even though the markers used in lineage classifications were part of these analyses [24]. Another recent study that assessed 47 potential markers for use in *C. cayetanensis* genotyping also observed that phylogenies based on these markers using data from available genomes did not demonstrate isolate segregation based on lineage assignment, although the lineage typing markers employed by this study did not include the lineage typing markers used by the CDC [23]. Additionally, phylogenetic analyses between USDA_Mex32 and 20 other *C. cayetanensis* isolates based on just lineage typing loci did support the association of lineage A isolates, but support of lineages B and C was dependent on analysis with both ML and NJ trees not supporting a clear segregation of these lineages (Fig. 2). Clearly the field of *C. cayetanensis* genotyping is in a state of rapid change with the best markers for genotype detection and discrimination remaining to be fully defined. As more isolates are sequenced and become available, future analyses may help to clarify the relationships between different strains and lineages of *C. cayetanensis* with more and better genomic data being an important part of resolving these discrepancies.

## Conclusions

Clearly more genomic data are needed to improve our ability to analyze differences between isolates, strains, and lineages of *C. cayetanensis*. To perform such analyses, more genomes from Mexico and other regions of the world will be required. Methods for sample preparation, sequencing, and assembly that can extract quality genomic data from the often-low quantities of starting material that are present in such isolates will be essential to advance the field of *C. cayetanensis* genomics.

### Electronic supplementary material

Below is the link to the electronic supplementary material.

Additional file 1

Additional file 2

Additional file 3

## Data Availability

All data generated or analyzed during this study are included in this published article and its supplementary information files; genome assemblies and raw sequences are available at NCBI under the BioProject number PRJNA1045665.

## List of abbreviations

U.S.: United States
CDC: Centers for Disease Control and Prevention
NCBI: National Center for Biotechnology Information
ARS-USDA: Agricultural Research Service-United States Department of Agriculture
USDA_Mex32: Strain name given to isolate used in this study
