# Supplementary figures and images for: The first Cyclospora cayetanensis lineage A genome from an isolate from Mexico
Source: BMC Genomics. 2024 Mar 5;25:246. doi: 10.1186/s12864-024-10163-y (PMC10913667; doi:10.1186/s12864-024-10163-y)

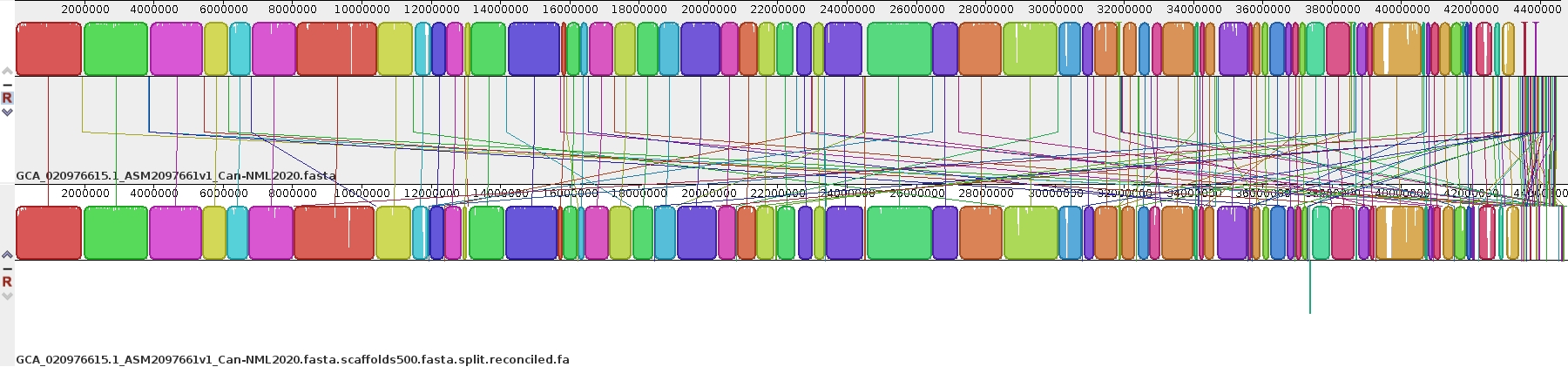

Supplement: Supplementary file 2 — Additional file 2 [file 12864_2024_10163_MOESM2_ESM.jpg]

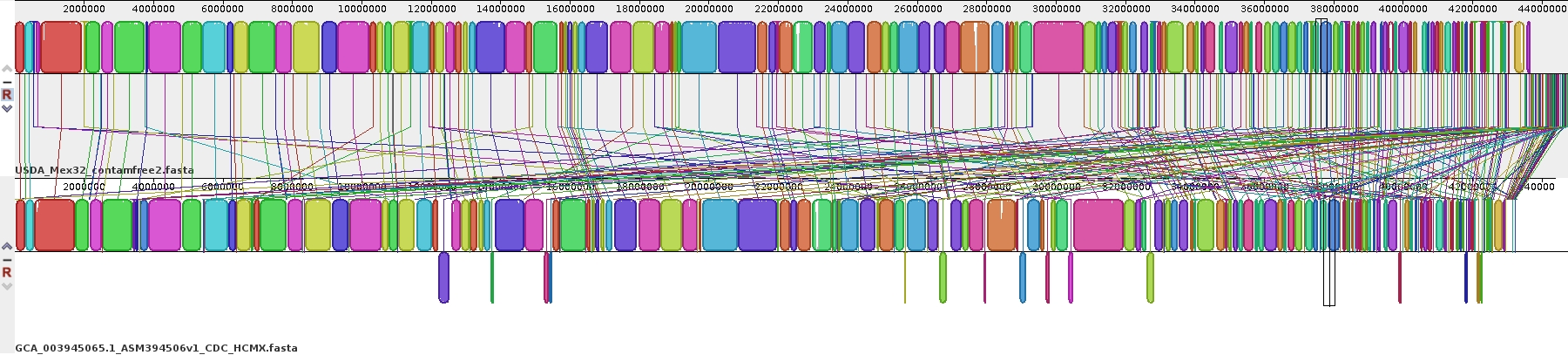

Supplement: Supplementary file 3 — Additional file 3 [file 12864_2024_10163_MOESM3_ESM.jpg]
